# Interleukin-17-positive mast cells influence outcomes from BCG for patients with CIS: Data from a comprehensive characterisation of the immune microenvironment of urothelial bladder cancer

**DOI:** 10.1371/journal.pone.0184841

**Published:** 2017-09-20

**Authors:** Alexander C. Dowell, Ellen Cobby, Kaisheng Wen, Adam J. Devall, Vinnie During, Jane Anderson, Nicholas D. James, Kar K. Cheng, Maurice P. Zeegers, Richard T. Bryan, Graham S. Taylor

**Affiliations:** 1 Institute of Immunology & Immunotherapy, University of Birmingham, Vincent Drive, Birmingham, United Kingdom; 2 Royal Shrewsbury Hospital, Shrewsbury, Shropshire, United Kingdom; 3 University Hospitals Birmingham NHS Foundation Trust, Birmingham, United Kingdom; 4 Birmingham Clinical Trials Unit (BCTU), Institute of Applied Health Research, Public Health Building, University of Birmingham, Edgbaston, United Kingdom; 5 School of Health and Population Sciences, University of Birmingham, Birmingham, United Kingdom; 6 Nutrition and Translational Research in Metabolism (School NUTRIM), and Care and Public Health Research Institute (School CAPHRI), Maastricht University, The Netherlands; 7 Institute of Cancer and Genomic Sciences, University of Birmingham, Birmingham, United Kingdom; Universita degli Studi di Palermo, ITALY

## Abstract

The tumour immune microenvironment is considered to influence cancer behaviour and outcome. Using a panel of markers for innate and adaptive immune cells we set out to characterise and understand the bladder tumour microenvironment of 114 patients from a prospective multicentre cohort of newly-diagnosed bladder cancer patients, followed-up for 4.33±1.71 years. We found IL-17-positive cells were significantly increased in primary and concomitant carcinoma in situ (CIS), p<0.0001, a highly malignant lesion which is the most significant single risk factor for disease progression. Further characterisation of the tumour immunophenotype identified IL-17+ cells as predominantly mast cells rather than T-cells, in contrast to most other tumour types. Expression of the IL-17-receptor in bladder tumours, and functional effects and gene expression changes induced by IL-17 in bladder tumour cells in vitro suggest a role in tumour behaviour. Finally, we assessed the effects of IL-17 in the context of patient outcome, following intravesical BCG immunotherapy which is the standard of care; higher numbers of IL-17+ cells were associated with improved event-free survival (p = 0.0449, HR 0.2918, 95% CI 0.08762–0.9721) in patients with primary and concomitant CIS (n = 41), we propose a model of IL-17+ Mast cells mechanism of action. Thus, in the context of bladder CIS, IL-17+ mast cells predict favourable outcome following BCG immunotherapy indicative of a novel mechanism of BCG immunotherapy in UBC and could form the basis of a stratified approach to treatment.

## Introduction

Bladder cancer is the seventh most common cancer in Western society, with a global incidence of over 380,000 [1,2]. In Western populations 90% of bladder cancers are transitional cell carcinoma of urothelial origin (urothelial bladder cancer, UBC) and most patients (75–85%) present with non-muscle invasive bladder cancer (NMIBC: stages Ta/T1/Tis) [3]. Patients with NMIBC are initially treated by transurethral tumour resection (TURBT), but recurrence is commonplace occurring in up to 80% of patients [4]. Progression to muscle-invasive bladder cancer (MIBC: stages T2+) occurs in up to 45% of patients [4,5], and represents a critical step in the disease course, carrying a 5-year survival rate of only 27–50%, necessitating more radical therapies (including surgery, chemotherapy or radiotherapy) [6,7].

The most significant single risk factor for progression to MIBC is the presence of primary or concomitant carcinoma in situ (CIS) [8]. This flat high-grade dysplasia is highly malignant with significant potential for invasion; patients diagnosed with CIS therefore undergo additional treatments following TURBT, principally repeated cycles of intravesical Bacillus Calmette-Guerin (BCG) immunotherapy in a regimen of induction and maintenance [9]. Despite these efforts, 50% of patients relapse and are then at high risk of progression to MIBC, with poor prognosis [10]. There are currently no prognostic markers to identify those CIS patients who will respond to therapy and those who will relapse [9].

The tumour microenvironment is important in the initiation, growth and progression of cancer, and multiple interactions between tumour, stromal and immune cells have been described [11]. The contribution made by immune cells is complex—many different cell types have been identified within tumours, and the effects of a particular infiltrate can vary between different tumours [6,7]. With regard to NMIBC, the potential role of the immune system is of particular interest since the most successful treatment currently utilised, BCG immunotherapy, is thought to act by inducing an acute inflammatory response in the bladder wall [12,13]. Studies thus far have examined macrophages [14], T cells [15,16] and the inflammatory response provoked by BCG [17], but many questions remain unanswered [12]. Therefore, our objective was to comprehensively characterise the immune microenvironment of UBC, and its influence on outcomes, utilising tumour material prospectively collected from newly-diagnosed patients [18].

## Material and methods

### Patient samples

Formalin fixed paraffin embedded (FFPE) tissue and snap-frozen tissue samples of newly-diagnosed primary UBCs were obtained from the Bladder Cancer Prognosis Programme (BCPP—clinicaltrials.gov identifier NCT00553345, ethics approval 06/MRE04/65) [18]. Collection was performed at initial TURBT, prior to adjuvant treatment, as previously described [18]. Patients provided informed written consent to have data from their medical records used in research (UK Research Ethics Committee approval: 06/MRE04/65). Patient demographic information is presented Table A in S1 File.

### Immunohistochemistry

Full description of immunohistochemistry protocols is provided in S2 File. Briefly, after de-waxing and antigen retrieval, FFPE bladder tumour sections were stained with a range of antibodies (Table A in S2 File) and HRP-DAB and/or alkaline phosphatase-vector red enzyme-substrate combinations (Vector, UK), with haematoxylin (Sigma, UK) counterstain. Images are at x100 magnification unless otherwise stated.

### Staining quantification

Slides were assessed by a qualified pathologist (EC), blinded to the hypothesis being tested. For each patient, numbers of positive cells in 10 high power fields (field diameter of 0.50mm) were counted. Peritumour was defined as the adjacent area within one high power field from the tumour. Stroma was defined as more than one high power field from tumour. Slides that did not contain tumour were omitted.

### Cell lines, growth and migration assays

Bladder cancer cell lines 5637 and HB-CLS-2 were obtained from the ATCC. EJ cells were provided by Dr. N. Shimwell, University of Birmingham. The breast cancer cell line MDA-MB-231 was provided by Dr. R. Grand, University of Birmingham. Cell lines were cultured in RPMI-1640 medium supplemented with 10% foetal bovine serum, 100 U/ml penicillin and 0.1 mg/ml streptomycin (Sigma); mycoplasma testing was carried out on receipt and at regular intervals. Recombinant human IL-17A (Peprotech, UK) was added as described. Cell growth was measured following 5 day culture in 96 well plates using WST assay (Clontech, USA). Following the manufacturer’s instructions WST was added and assayed after 2 hours using a Wallac Victor2 1420 Multilabel counter (PerkinElmer, Italy). Cell migration was measured using a standard wound healing assay: cells in triplicate wells of a 24 well plate at 1x10^5^ cells/well with rhIL-17 added for 48hrs before wounding using a pipette tip. Following wounding, cells were washed and new media with or without IL-17 added as appropriate. Photographs were taken at 0h and 6h post-wounding and the area of the wound calculated using SPOT Image software (SPOT Imaging Solutions, USA).

### ELISA and flow cytometry

IL-6 and IL-8 were measured by ELISA (BioLegend UK Ltd) following the manufacturer’s protocol. Cell surface levels of the IL-17 receptor (IL-17R) were measured by flow cytometry of cells stained with fluorophore-conjugated IL-17R antibody (BioLegend UK Ltd) performed on an Accuri C6 flow cytometer (BD Biosciences).

### RNA extraction and analysis

As described in S2 File, cells or tissue were homogenised in Trizol (Invitrogen) and isolated with an RNeasy kit (Qiagen). mRNA was quantified by q-RTPCR using Taqman probes (Applied Biosystems) listed in Table C in S2 File. A Human Genome U133 Plus 2.0 Array (Affymetrix) was used to assess global gene expression changes in EJ or 5637 cells untreated or treated with IL-17. A change in gene expression of 1.5 fold was used to dictate inclusion.

### Statistical analysis

Data was analysed using the Graphpad Prism program (Graphpad Software Inc., USA). Statistical tests used are detailed for each result. For survival analysis, time to event was calculated from initial registration to either first event, death or last known attendance at clinic.

## Results

### The immune microenvironment of bladder cancer

**Fig 1:**
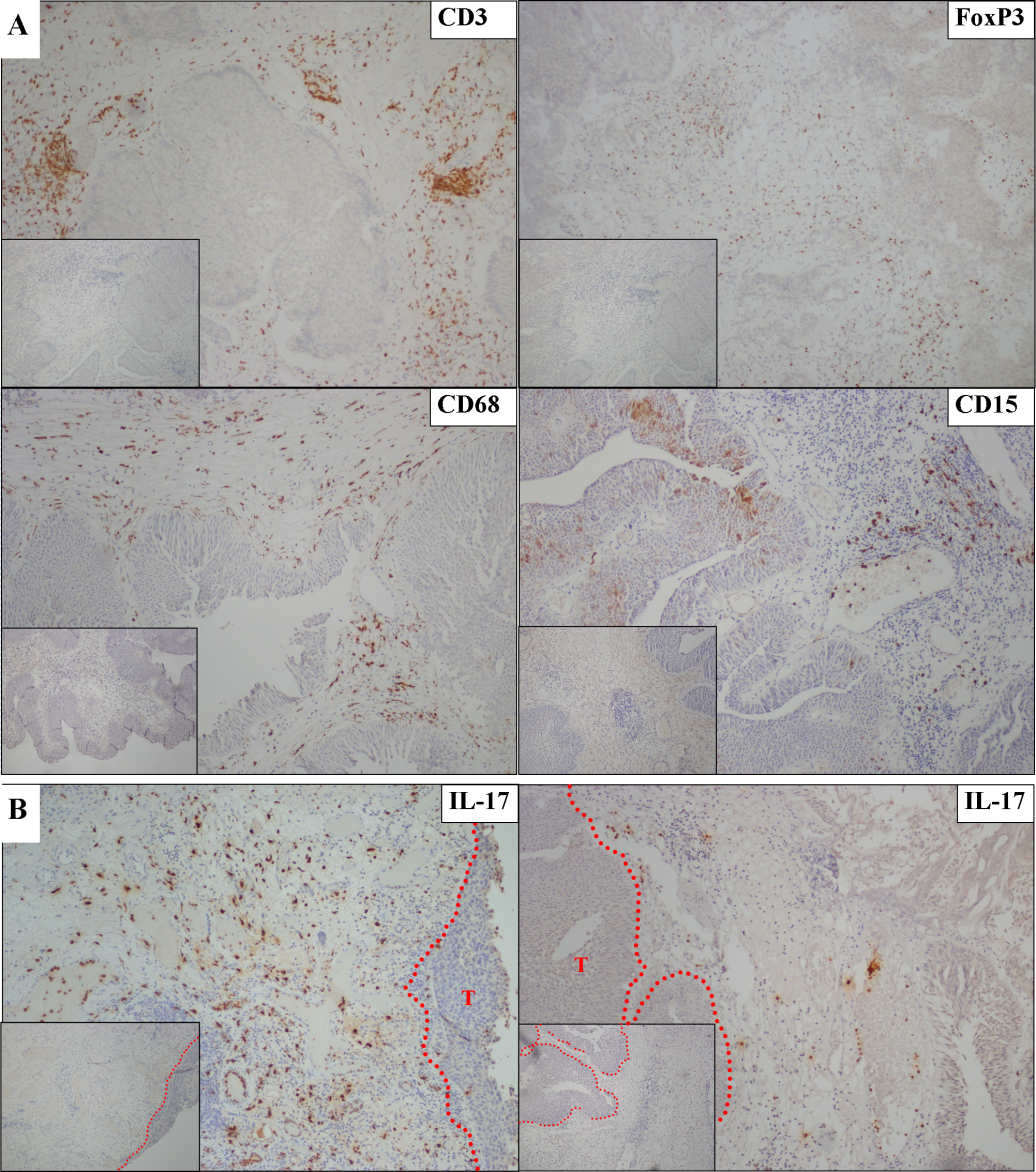
Immunohistochemical analysis of the bladder cancer immune microenvironment. Representative results from bladder cancer FFPE biopsy sections stained using primary antibodies specific for the indicated immune cell markers. Positive cells are stained with DAB (brown) and all slides are counterstained with haematoxylin (blue). Negative control images (inset) were obtained by substituting an isotype control antibody for the primary antibody. **A:** The bladder cancer microenvironment contains CD3+ T cells, FoxP3+ cells, CD68+ macrophages and CD15+ granulocytes. **B**: Representative images from two bladder cancer biopsies with high (left) or low (right) numbers of IL-17 positive cells present. Note the different distributions of IL-17+ cells and CD3+ cells shown in A.

We first examined the frequency of immune cells present in the microenvironment of UBC using a panel of antibodies specific for markers of the adaptive, (CD3, T cells; FoxP3, regulatory T cells) and innate (TCRγ, γδT cells; CD15, granulocytes; CD68, macrophages) immune responses. Representative examples of this analysis are shown in Fig 1. Infiltrates of CD3+ T-cells were mostly present in lymphoid aggregates. Lower numbers of FoxP3+ cells were also present with a similar distribution. In contrast, no γδT-cells were present despite reliably detecting these cells in control tonsillar tissue (S1 Fig). In keeping with previous reports [14], large numbers of CD68+ macrophage were present and these were more widely distributed throughout the tumour than T cells. Interestingly, we found CD15+ granulocytes present within the bladder cancer microenvironment with a similar distribution to macrophages.

An antibody specific for the cytokine IL-17 was included in our panel. This cytokine is widely attributed to Th17 cells, a subset of CD4+ T cells, although other immune cells also produce IL-17 [19]. Increased numbers of Th17 cells are associated with both improved and poorer outcomes dependent on the disease setting [20–24]. IL-17 has been implicated in the response to BCG immunotherapy in a murine bladder cancer model [25]; however, there are no data from patients as to the role of IL-17 in UBC. As shown in Fig 1B, there was noticeable variation in IL-17 positive (IL-17+) cells in different tumour biopsies. When present, the majority of IL-17+ cells did not infiltrate into the tumour but were located in the tumour microenvironment either peritumorally or in the surrounding tumour stroma.

### IL-17+ cells are increased in carcinoma in situ

**Fig 2:**
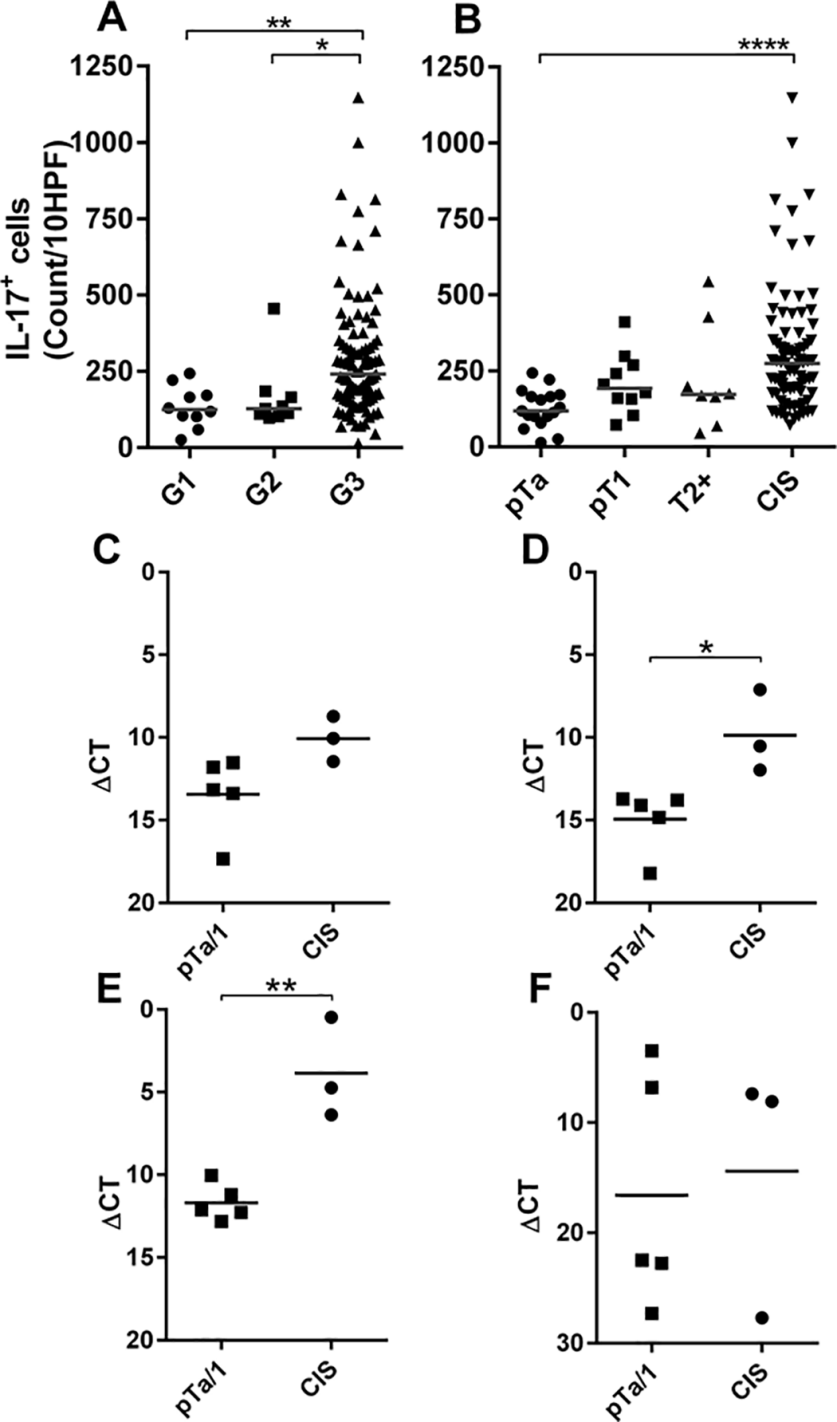
Association between IL-17 positive cells and bladder cancer grade and stage. **Upper panels:** The number of IL-17 positive cells present in different bladder cancer biopsies are displayed according to (**A**) grade at time of diagnosis (G1, n = 10; G2, n = 9; G3 n = 95) or (**B**) stage at time of diagnosis (pTa, n = 17; pT1, n = 10; T2+, n = 8; CIS, n = 83). Horizontal bars indicate the mean number of cells present in all biopsies within each subgroup. **Lower panels:** Results of quantitative rtPCR measuring RNA transcripts present in whole snap frozen biopsies diagnosed as stage pTa/pT1(n = 5) or pTa/pT1 with concomitant CIS (n = 3). The ΔCT values of mRNA transcripts of (**C**) IL-17A, (**D**) IL-17-F, (**E**) IL-6 or (**F**) IL-23 are shown relative to GAPDH (ΔCT = CT_Experimental_-CT_GAPDH_). Asterisks indicate significance calculated by one way ANOVA with Dunn’s multiple comparison test (B) or unpaired T-test (C-F): *p<0.05, **p<0.01, ****p<0.0001, n/s = not significant.

The marked differences observed in the frequency of IL-17+ cell infiltrate led us to stain more tumour specimens (n = 48). All biopsy samples with elevated IL-17+ cells were from patients with concomitant CIS; therefore, we expanded our analysis to include further CIS patients (n = 83). Regarding all patients, all but one sample with elevated numbers of IL-17+ cells were from grade 3 tumours; however, not all grade 3 tumours had elevated numbers of IL-17+ cells (Fig 2A). Examining the stage of the tumours at time of diagnosis demonstrated that there was a significant increase in IL-17+ cells in CIS patients (p = <0.0001), although not all CIS patients have enhanced levels of IL-17+ cells (Fig 2B).

We next performed qRT-PCR to confirm that the IL-17+ cells detected in CIS biopsies were expressing the cytokine *in situ*. Ideally, qRT-PCR would have been performed after isolating IL-17+ cells by laser capture microdissection, allowing the transcriptome of the IL-17+ cells to be examined. Unfortunately, no useable RNA could be extracted from microdissected cells after IL-17 staining. We therefore obtained whole tumour biopsy RNA from patients with NIMBC grade 3 disease with or without concomitant CIS and examined gene expression in the total tumour microenvironment. Even though tumour and stromal cells were present in excess, we detected increased IL-17 mRNA transcripts in samples from patients with CIS (Fig 2C), concordant with the staining data, although these data did not reach significance. Examining transcripts related to and downstream of IL-17, we similarly measured increased mRNA levels of the related cytokine IL-17F as well as IL-6, the expression of which is upregulated by IL-17 [26] (Fig 2D and 2E). Interestingly, there was no difference in levels of IL-23 mRNA, a cytokine that promotes IL-17 secretion and maintains Th17 memory CD4+ T cells [27,28].

### Direct effects of IL-17 on bladder cancer cells in vitro

**Fig 3:**
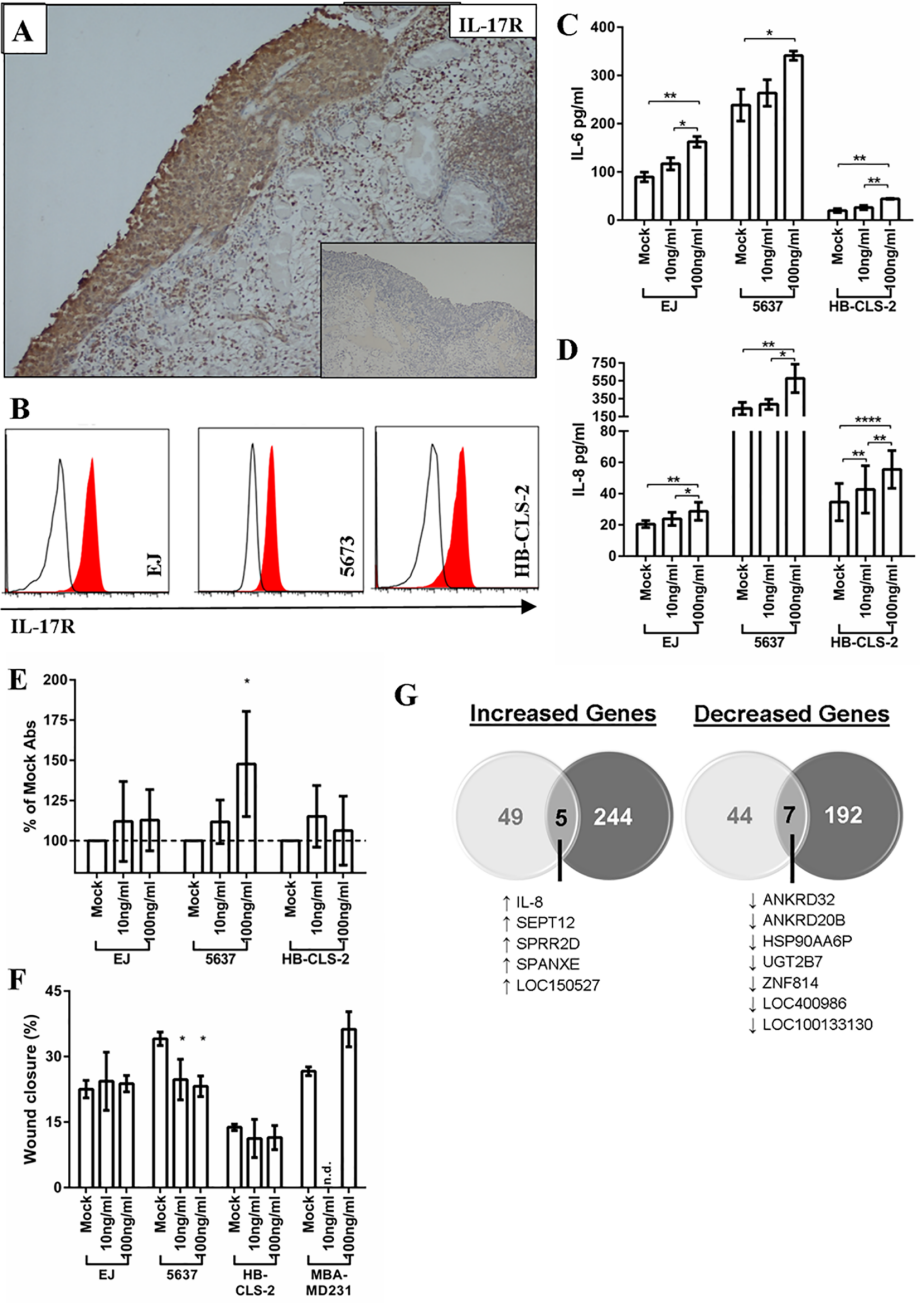
IL-17 receptor expression and function in bladder cancer. **A:** Representative result obtained for a bladder cancer biopsy, in this case CIS, stained with a primary antibody specific for the IL-17 receptor (brown staining represents receptor expression). Inset image shows result obtained using an isotype control primary antibody. **B:** Histograms showing levels of IL-17 receptor on surface of the urothelial cell lines EJ, 5637 or HB-CLS-2 measured using flow cytometry. Red histogram, anti-IL-17-receptor antibody; open histogram, isotype control antibody. **C & D:** IL-17 treatment increases production of IL-6 and IL-8 by all three urothelial cell lines tested. Cytokine levels were measured by ELISA. Error bars represent the standard deviation of the mean, which was calculated from three (IL-6) or four (IL-8) independent experiments. Asterisks indicate significance calculated by repeated measure ANOVA with Bonferroni multiple comparison test: *p<0.05, **p<0.01, ***p<0.001.**. E:** Growth of EJ, 5637 and HB-CLS-2 cells after five days incubation in the indicated amounts of IL-17. Cell growth was measured by WST assay. Four independent experiments were performed and the mean change in WST-1 absorbance relative to mock treated cells is shown. Error bars indicate standard deviation. **F:** Migration of the above cells in a 6 hour wound healing assay in the absence or presence of the indicated concentrations of IL-17. Cells were grown in IL-17 for 48 hours before wounding. The breast cancer cell line MBA-MD231, which is more invasive in response to IL-17, was included as a positive control. Three independent experiments were performed and the mean wound closure is shown, error bars represent standard deviation. In both panels: *p<0.05 calculated by a repeated measure ANOVA with Bonferroni multiple comparison test. **G:** Venn diagrams showing gene expression changes induced by IL-17 treatment of EJ cells (light grey circles) or 5637 cells (dark grey circles) measured in a gene array. The number of genes that undergo similar changes in expression following IL-17 treatment are shown in the intersect, and are also listed below each Venn diagram.

We next considered the potential biological consequences of elevated numbers of IL-17+ cells. IL-17 has been reported to act directly on tumour and stromal cells that express the IL-17 receptor (IL-17R). To our knowledge IL-17R expression had not been examined in UBC and, consequently, the potential direct effects of IL-17 were unknown. We therefore undertook two sets of experiments to determine the effect of elevated IL-17 on bladder tumours. First, we stained 14 bladder cancer biopsies (randomly selected to represent different disease stages) with an IL-17R-specific antibody. All were positive for IL-17R irrespective of the tumour grade or stage (Fig 3A); this finding was subsequently confirmed by others during the preparation of this manuscript [29]. Next, we examined the direct effect of IL-17 on three bladder cancer cell lines (EJ, 5637 and HB-CLS-2) all of which expressed IL-17R at the same level (Fig 3B). All three bladder cancer cell lines were responsive to IL-17, as demonstrated by increased production of IL-6 and IL-8 (Fig 3C and 3D). However, subsequent experiments revealed marked differences in the way these cell lines responded to IL-17: growth of 5637 cells was increased by IL-17 in a dose dependent manner, yet in the same experiments growth of EJ or HB-CLS-2 cells was not significantly altered (Fig 3E). A similar pattern of results was seen in wound healing assays with 5637 cells showing a significant response to IL-17 resulting in decreased migration, while EJ and HB-CLS-2 cells were unaffected (Fig 3F). These findings are in contrast to previous work on other cancer cell lines showing IL-17 to enhance cell migration [24], as demonstrated by the breast cancer control cell line included in our experiments (MDA-MB-231).

### Direct effects of IL-17 on gene expression in vitro

The marked differences in phenotype exhibited by different bladder cancer cell lines exposed to IL-17 led us to perform microarray analysis to examine the effects of IL-17 on global gene expression. The 5637 and EJ cells that demonstrated contrasting behaviour in the functional assays above were either treated or untreated with IL-17 as before and changes in gene expression were analysed. Both cell lines responded to IL-17 but with notably different changes in gene expression (Fig 3G). Thus, for 5637 cells 448 genes was altered by IL-17 (249 upregulated, 199 downregulated) and 105 genes were altered for EJ cells (54 upregulated and 51 downregulated). Comparing both cell lines, only 12 genes were altered in the same way by IL-17 (5 upregulated, 7 downregulated). One of the upregulated genes was IL-8, the increased production of which had been observed earlier following IL-17 treatment (Fig 3D). Furthermore, in agreement with earlier experiments, the data showed IL-17 to increase expression of IL-6 in both cell lines; this gene was not included as one of the upregulated genes because although the increase in EJ cells (1.93 fold increase) was above the pre-defined 1.5-fold cut-off used to score response, the increase for 5637 cells fell marginally below (1.38 fold increase). We performed cluster analysis on the genes that were upregulated in 5637 cells by IL-17 (Table B in S1 File). Genes associated with growth factor activity were increased and significantly enriched (a result in-keeping with the increased growth of these cells following IL-17 treatment); genes involved in angiogenesis were also upregulated by treatment with IL-17. In contrast, genes involved in the inflammatory response and cell adhesion were downregulated by IL-17 treatment.

### Mast cells constitute the majority of IL-17 expressing cells in CIS

The Th17 subset of CD4+ T cells are recognised as important producers of IL-17, although other immune cells also produce IL-17 [26]. Two observations suggested that the IL-17+ cells present in CIS samples were not CD4+ Th17 cells. First, the morphology of the cells staining positive for IL-17 in the biopsies did not appear to be lymphocytic. Second, we noted the general localisation of IL-17+ cells was different to CD3+ cells in serial sections from the same CIS biopsy (Fig 1).

**Fig 4:**
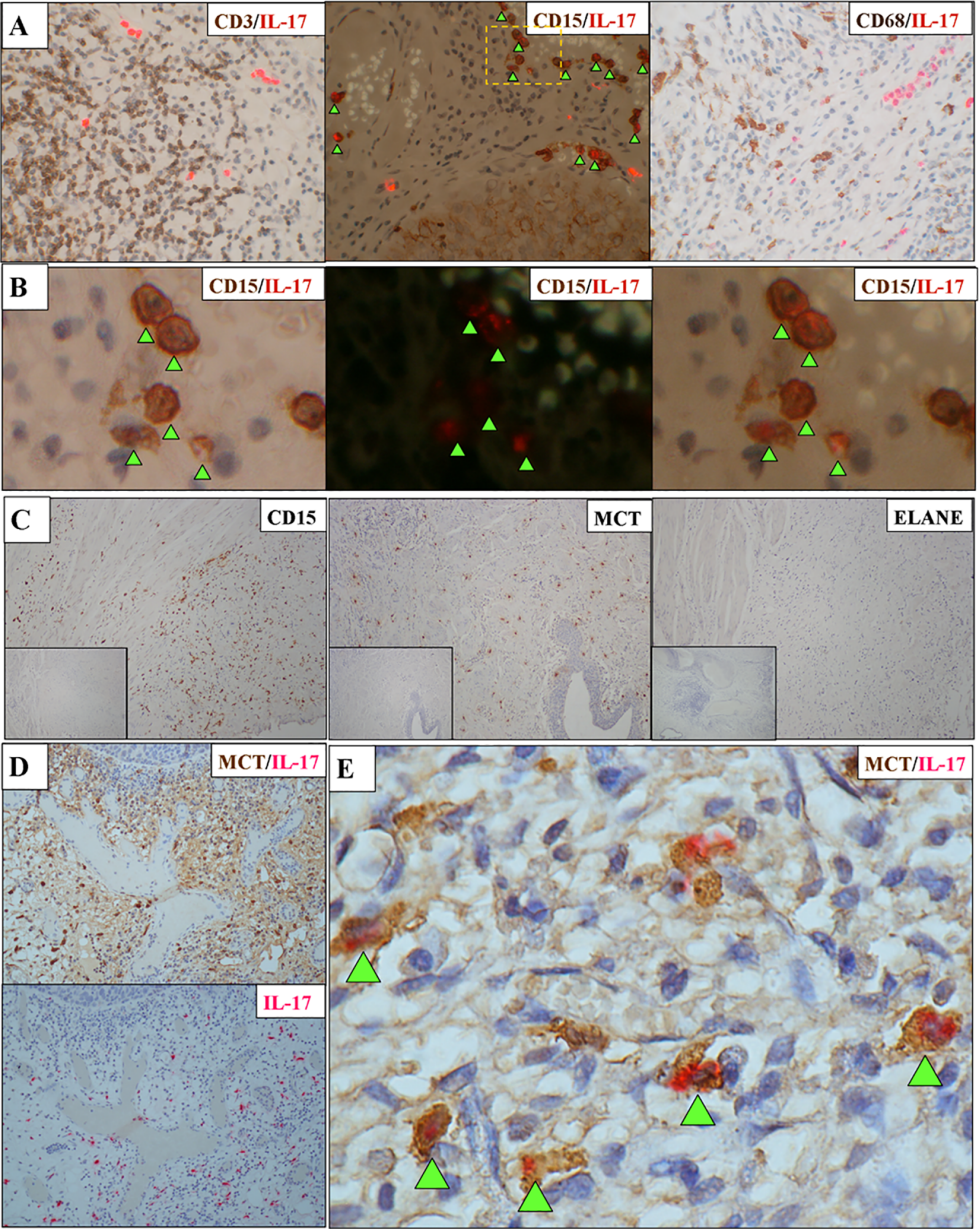
Identification of the IL-17 producing cells present in CIS. **A:** Representative results of co-staining for IL-17 and cell lineage markers (combined IF and bright field images. Red fluorescence indicates IL-17 and brown staining indicates CD3 (left image), CD15 (middle) or CD68 (right image). Note that CD15 and IL-17 co-localise as indicated by arrows. **B:** The region indicated by dashed lines in the IL-17/CD15 co-staining image above is magnified and shown in bright field (left), IF (middle) or combined IF/bright field (right). Arrows indicate the location of co-staining cells visualised in the combined IF/bright field image. **C:** Characterising the CD15-positive cells present in CIS. Representative staining results from a single CIS biopsy containing a large number of CD15 positive granulocytes (left panel). This biopsy also contains MCs that stain positive for MC tryptase (MCT). No ELANE-positive neutrophils were present (right panel). In each image positive cells are stained brown. The total number of biopsies stained for CD15, ELANE or MCT were 15, 18 and 12 respectively. The inset images show sections stained using appropriate isotype control primary antibodies. **D:** Co-localisation of MCT and IL-17 staining in CIS biopsies. Upper image shows the result obtained for a CIS biopsy co-stained with anti-IL-17 and anti-MCT antibodies (red and dark brown staining respectively). Note how the weaker IL-17 signal is obscured by the dark brown staining of the MCT-positive cells. Lower image shows a serial section from the same tumour specimen stained for IL-17 alone (red) for comparison. The result is representative of eight CIS biopsies analysed. **E:** Result of co-staining a CIS biopsy for IL-17 (red) and reduced intensity MCT (brown). Arrows indicate MCT-positive MCs that co-stain for IL-17 (magnification x1000). Image is representative of the four CIS biopsies stained using the modified protocol.

To identify the IL-17+ cell type in bladder CIS, we optimised the immunostaining protocols to allow FFPE biopsies to be stained for IL-17 and co-stained for CD3 (to identify T cell subsets), CD68 (to identify macrophages) or CD15 (to identify granulocytes). As expected, we did not see significant co-localisation of IL-17 with CD3, nor did we see co-localisation with CD68 (Fig 4A). However, the majority of IL-17+ cells were also positive for CD15 (Fig 4A and 4B), a molecule present on the surface of all granulocytes including neutrophils, mast cells (MC) and a subset of myeloid derived suppressor cells (MDSCs). Because these 3 cell types have each been reported to produce IL-17 [26] we stained sections for specific markers of neutrophils (ELANE) or MCs (mast cell tryptase, MCT). We saw little or no ELANE staining but large numbers of MCT-positive cells were present (Fig 4C and S2 Fig). To formally show that the IL-17+ cells were indeed MCs, we undertook a final series of co-immunostaining experiments. Immunostaining of IL-17 was predominantly lost when sections were co-immunostained for MCT (Fig 4D), consistent with strong cytoplasmic MCT staining masking the IL-17 signal. Interestingly, a small number of IL-17+ cells could still be observed on these slides, suggesting that the biopsies contained a small number of non-MCs that also produced IL-17. To formally demonstrate IL-17 and MCT co-localisation we repeated the co-immunostaining experiments, modifying the protocol to reduce the intensity of MCT staining. As shown in Fig 4E, cells positive for both MCT and IL-17 were observed in all cases confirming that MCs are the main source of IL-17 in the tumour microenvironment of bladder CIS.

### Elevated numbers of IL-17+ cells correlate with improved BCG response in CIS

**Fig 5:**
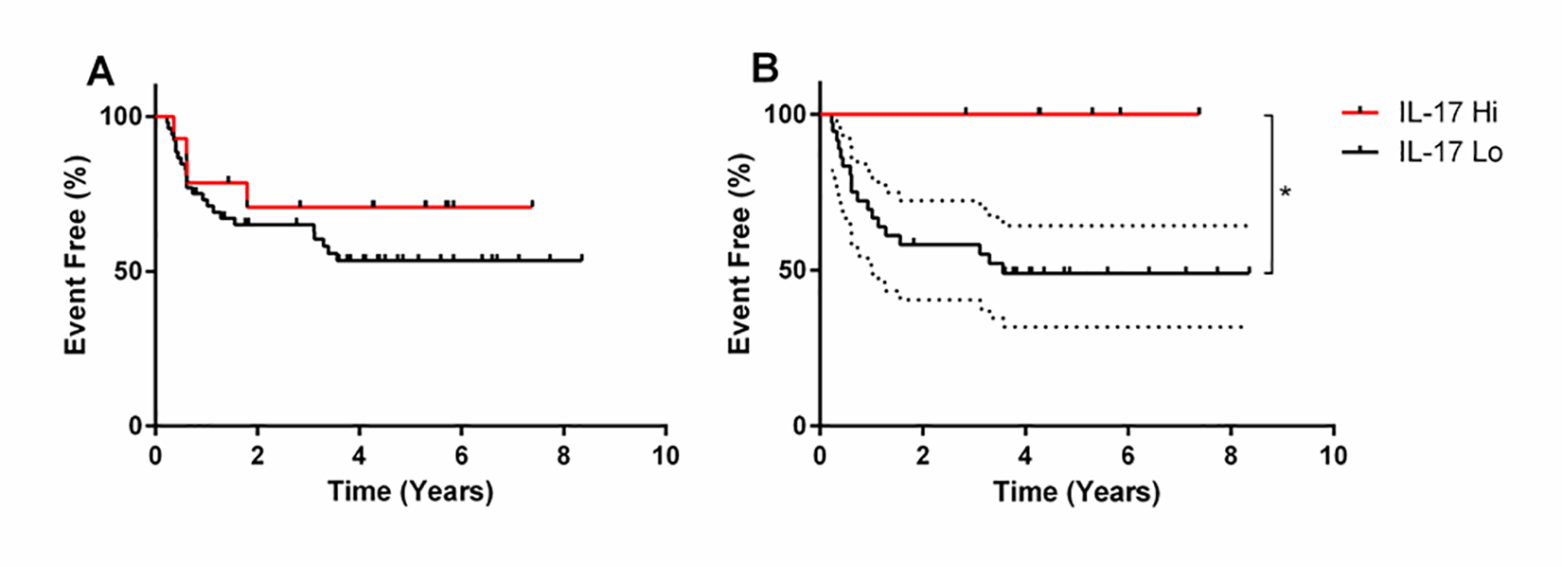
The effect of tumoral IL-17 positive cells upon patient outcome. **A:** Patients who had NMIBCs with concomitant CIS were divided into IL-17hi (n = 14) and IL-17low (n = 52) groups based on the upper quartile of IL-17 positive cell counts in this group of patients (374.5 IL-17 positive cells per 10 high power fields). **B:** Patients who received BCG immunotherapy were divided into IL-17hi (n = 6) and IL-17lo (n = 36) groups, using the same criteria as before. Significance was calculated using the Log-rank (Mantel-Cox) test. Dotted line indicates 95% CI.

The wide variation in the number of IL-17+ cells in CIS specimens (Fig 2B) prompted us to examine whether there was an association between IL-17 status and outcome. Patients in the upper quartile of IL-17 positivity (above 374.5 cells per 10 hpf) did not initially seem to demonstrate different outcomes to the remainder (Fig 5A). However, IL-17 has been implicated in the efficacy of BCG immunotherapy in a murine bladder cancer model [25]; although patients with high grade NMIBC should be offered intravesical BCG, not all patients actually receive this treatment. Therefore, we examined the outcome of patients who actually received intravesical BCG (n = 41, Table C in S1 File). Using the same cut-off to divide patients into IL-17 high and low (above or below 374.5 cells per 10 hpf, respectively), there was a significant difference in outcome (Fig 5B): patients with high numbers of IL-17+ cells had better event-free survival (p = 0.0449, HR 0.2918, 95% CI 0.08762 to 0.9721) when treated with BCG. These data strongly suggest that the presence of increased IL-17+ cells (the majority of which are mast cells) is beneficial for NMIBC patients with concomitant CIS when receiving intravesical BCG.

## Discussion

The tumour microenvironment is critically important in the development, progression and control of cancer [11]. Our aim was to further understand the immune microenvironment of UBC, making use of tumour samples collected at the time of diagnosis, prior to treatment. We detected a diverse infiltrate of innate and adaptive immune cells including macrophages and T cells (which have been described by others [14,30]), and also FoxP3+ Tregs [16]. Interestingly, we did not detect infiltration of γδT cells, a cell type reported to inhibit tumour growth in a murine orthotopic bladder cancer model [25]. The most novel finding was the marked variation in the numbers of IL-17+ cells between different patients’ tumours, with increased numbers of these cells significantly associated with CIS. However, we also observed that some patients with CIS had few IL-17+ cells in their tumours, suggesting their presence was more complex than a simple causal association.

We next examined the identity of the IL-17+ cells we observed in CIS. Based on their appearance, frequency and distribution within the tumour, the IL-17+ cells were clearly not T cells. Their expression of CD15 suggested they were neutrophils, MDSCs or MCs. The crucial observation that almost all IL-17+ cells co-localised with MCT, a highly-specific MC marker, highlighted MCs as the predominant source of IL-17 in bladder CIS.

The increased frequency of IL-17+ MCs in CIS raised the question of their role. MCs have been reported to have anti-tumour effects, modifying anti-tumour immune responses and enhancing T cell recruitment [31,32]. Conversely, MCs enhance tumorigenesis and angiogenesis [33–35], and have been reported to inhibit anti-tumour immunity [36,37]. Likewise, the role of IL-17 in cancer is complex. In IL-17 knockout mice some tumours grow more rapidly, whilst others grow more slowly, suggesting that IL-17’s effects are context dependent [38]. This is borne out in the clinical setting with reports of both better and worse outcomes associated with increased numbers of Th17 cells [20,22,39–41]. To our knowledge, no-one has previously studied the effects on outcome in the UBC setting.

Although IL-17 can act indirectly (for example, promoting tumour growth by altering the immune microenvironment [21]), it can also act directly upon tumour cells that bear IL-17R [42]. Since we found that IL-17R was expressed by many bladder tumours, we decided to focus on the potential direct effects of IL-17 on bladder cancer cells. IL-17 induced differential responses, demonstrated by the specific changes in phenotype and gene expression in different cell lines. IL-17 increased the growth rate of 5637 cells, concurring with previously published work examining the MB49 murine bladder cancer cell in mice [42]. Furthermore, IL-17 treatment of 5637 cells increased the expression of genes involved in angiogenesis and decreased the expression of those involved in cell adhesion. The former could potentially contribute to increased tumour growth *in vivo*, while the latter is consistent with CIS being a loosely-adherent lesion that often disseminates by the shedding and re-implantation of cells [43]. In contrast, growth of the other two bladder cancer cell lines that we studied was not significantly affected by IL-17, despite eliciting a response by increased production of IL-6 and IL-8. Gene expression analysis revealed that the 5637 and EJ cell lines had very different patterns of gene expression in response to IL-17. Taken together these results demonstrate that the direct effects of IL-17 vary markedly, even within the context of a single tumour type.

Although these in vitro effects were interesting, we sought to identify the consequences of IL-17+ MCs in patients by utilising the long-term outcome data linked to the tumour samples. We acknowledge that such an approach cannot directly address biological mechanisms of action, but we argue that it is more relevant to human health since it takes into account the clinical interventions that patients undergo.

As IL-17+ MCs were significantly associated with CIS, a lesion with a poor prognosis, we anticipated that increased numbers of these cells would be associated with poorer outcome, an association recently observed in gastric cancer [23]. Paradoxically, CIS patients with higher numbers of IL-17+ MCs had significantly longer event-free survival following intravesical BCG treatment than patients with a lower number of cells. This finding is in-keeping with a recent study in oesophageal squamous cell carcinoma where IL-17+ MCs were also identified, and increased numbers significantly associated with favourable survival [44]. In our study, we only observed a significant effect of IL-17+ MCs in patients who underwent intravesical BCG treatment. The significant effect that we observed in CIS is seemingly larger than the survival advantage seen in oesophageal cancer [44]; one possibility, therefore, is that BCG amplifies the beneficial effects associated with increased numbers of IL-17+ MCs.

Currently, we can only speculate as to why some cases of bladder CIS demonstrate increased numbers of IL-17+ MCs and others do not. One possibility may be differences in the tumour microenvironment. MCs enter tissues in an immature form, maturing in response to local stimuli. Reports have demonstrated IL-17+ MCs at sites of active inflammation in rheumatoid arthritis [45–47], psoriasis [48] and atherosclerotic plaques [49]. Furthermore, inflammatory mediators have been shown to stimulate IL-17 production from purified human MCs in vitro [45]. Thus, the microenvironment of CIS may stimulate MCs to produce IL-17. We note that CIS biopsies contain both IL-17+ and IL-17- MCs. The latter may reflect recent immigrants that have not yet developed an IL-17-producing phenotype, or a distinct subtype of MCs unresponsive to the environmental cues that stimulate production of IL-17. In this regard, mature connective tissue MCs, but not immature bone-marrow-derived MCs, have been shown to produce IL-17 following toll-like receptor-2 ligation [50].

**Fig 6:**
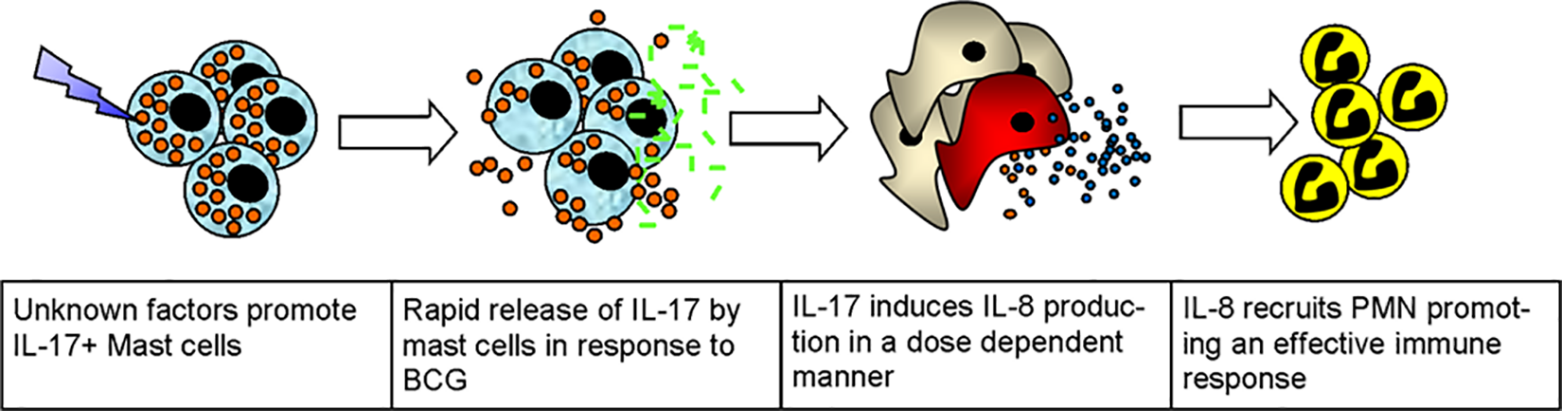
Proposed mechanism for the role of IL-17-positive cells in the efficacy of BCG immunotherapy.

Deeper understanding of the mechanisms responsible for the improved clinical outcomes associated with IL-17+ MCs and BCG is hampered by a lack of a suitable CIS mouse model [51,52], and fundamental differences between mouse and human MC biology. Many reports show IL-17 production by human MCs [19,23,45,47,48,53,54], but only a single study hitherto detected IL-17+ murine MCs, and this required culture of the cells in vitro [50]. Other studies suggest γδT cells produce IL-17 in place of murine MCs [19]; consistently, γδT cells produce IL-17 in response to BCG in a murine model of bladder cancer [25]. In this model, IL-17 was required for BCG efficacy through IL-8 production and increased neutrophil recruitment, in keeping with clinical data showing increased IL-8, within the first few hours of BCG treatment is predictive of response [12,55,56]. Since we have shown an absence of γδT cells in human bladder tumours, we propose an alternative scenario in humans. We suggest that the immediate response to BCG is elicited via MCs rapidly releasing IL-17, inducing IL-8 production by urothelium and, as we have shown, tumour cells recruiting increased numbers of neutrophils. It could then be envisioned that other immune cells, including γδT cells, will then be recruited, eliciting an improved immune response (Fig 6).

Recently, a urinary cytokine panel (CyPRIT), based on changes in levels of nine cytokines, correctly identified 85.5% of BCG responders in initial tests in 130 high-risk BCG-treated patients, of whom almost half had concomitant CIS [57]. Importantly, of the predictive cytokines, two are induced by IL-17 (IL-6 and IL-8), and another (TRAIL) is associated with neutrophils recruited by the IL-17/IL-8 axis, further supporting IL-17 as an important factor in the response to intravesical BCG.

Our observations are important for two reasons. Firstly, these data are indicative of a novel mechanism of action of BCG immunotherapy, which could be therapeutically targeted or manipulated, namely mimicking or enhancing the beneficial effects of increased IL-17+ MCs. This is a long-term aim that will require a more detailed understanding of whether IL-17+ MCs act as effectors or as surrogates of other factors, and confirmation of an event-free survival benefit in a larger study. This work is in progress. Secondly, if confirmed, our results have potential for immediate clinical utility since there are currently no routinely-used biomarkers to predict BCG responses in UBC patients. Incorporating IL-17 staining into standard histopathological tumour assessment, or potentially inclusion in CyPRIT, could be a simple way to stratify patients and guide clinical management. Such a stratified approach could reduce the unnecessary toxicity resulting from the futile use of BCG.

## Supporting information

S1 Fig Staining of bladder cancer or control tissue for γδT cells. Tissue sections were stained using a primary antibody specific for the T cell receptor gamma chain, appropriate secondary antibody and DAB. Slides were counterstained with haematoxylin. Shown is a representative result obtained for a bladder cancer biopsy section and a positive control tonsil tissue section.
(TIF)

S2 Fig Control experiment validating the ELANE staining protocol. A positive control tonsillar tissue section was stained for the neutrophil marker ELANE using appropriate antibodies and DAB. The slide was counterstained with haematoxylin. ELANE-positive cells are stained brown.
(TIF)

S1 File Table A in S1 File: Demographics of bladder cancer patients used to assess IL-17 cell infiltration. Table B in S1 File: Function of genes changed in 5637 cells following IL-17 treatment. Table C in S1 File: Detailed histories of BCG-treated CIS patient outcomes and treatment.
(DOCX)

S2 File Table A in S2 File: Details of antibodies used in immunohistochemistry. Table B in S2 File: Details of primers used for quantitative reverse transcriptase PCR.
(DOCX)
