# Supplementary figures and images for: Interleukin-17-positive mast cells influence outcomes from BCG for patients with CIS: Data from a comprehensive characterisation of the immune microenvironment of urothelial bladder cancer
Source: PLoS One. 2017 Sep 20;12(9):e0184841. doi: 10.1371/journal.pone.0184841 (PMC5607173; doi:10.1371/journal.pone.0184841)

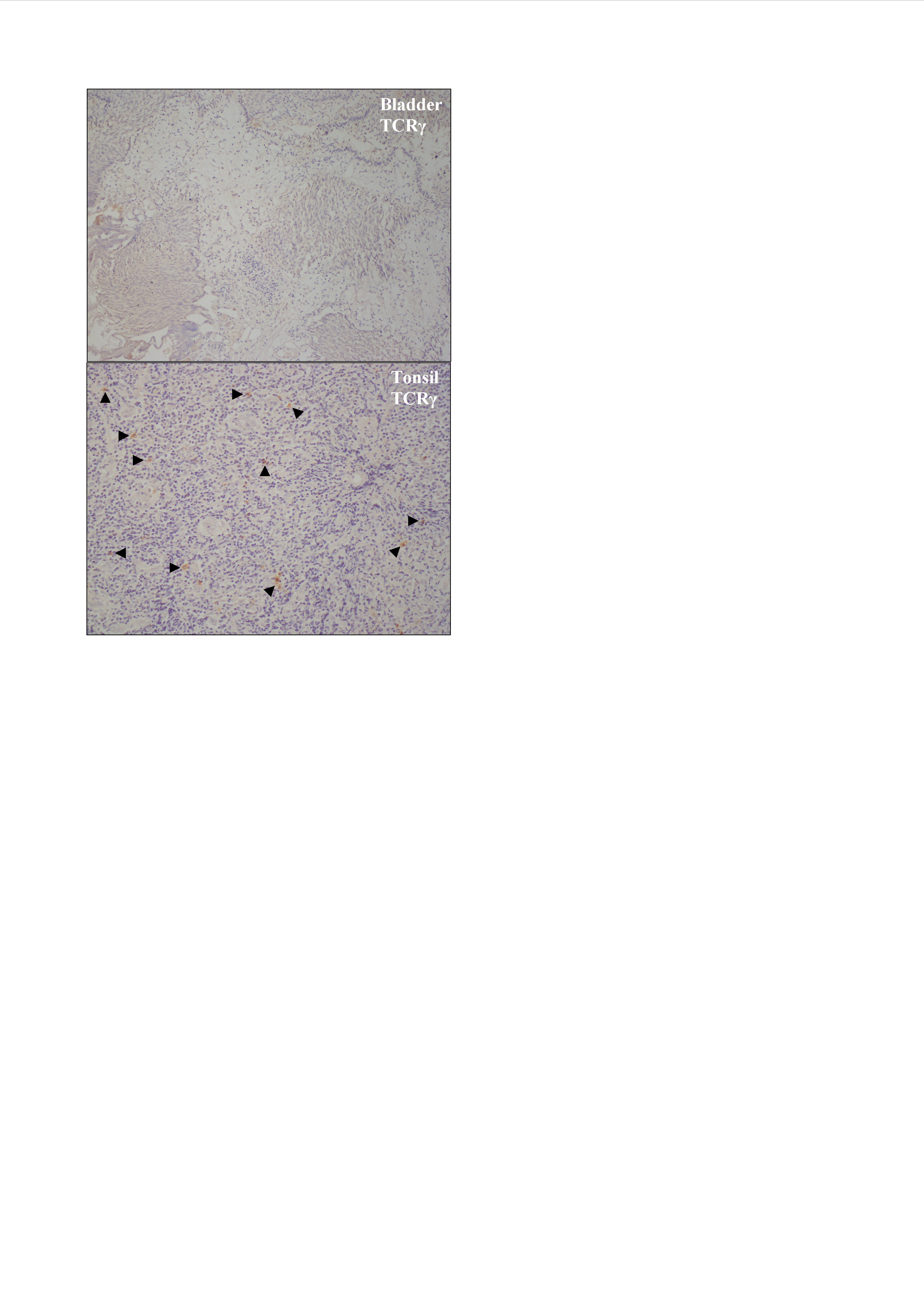

Supplement: S1 Fig — Tissue sections were stained using a primary antibody specific for the T cell receptor gamma chain, appropriate secondary antibody and DAB. Slides were counterstained with haematoxylin. Shown is a representative result obtained for a bladder cancer biopsy section and a positive control tonsil tissue section. (TIF) [file pone.0184841.s001.tif]

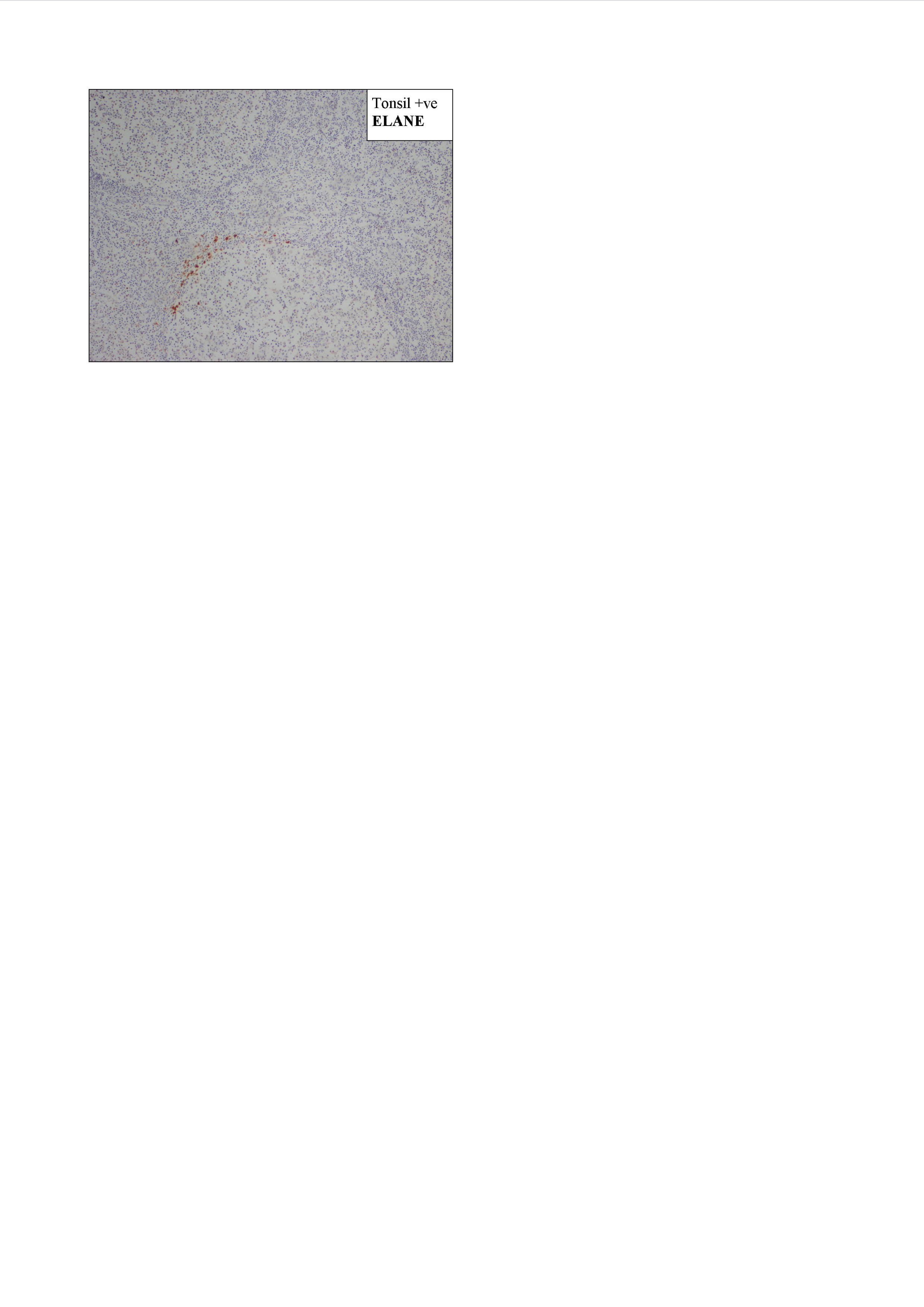

Supplement: S2 Fig — A positive control tonsillar tissue section was stained for the neutrophil marker ELANE using appropriate antibodies and DAB. The slide was counterstained with haematoxylin. ELANE-positive cells are stained brown. (TIF) [file pone.0184841.s002.tif]
