# Supplementary material for: Interleukin-17-positive mast cells influence outcomes from BCG for patients with CIS: Data from a comprehensive characterisation of the immune microenvironment of urothelial bladder cancer
Source: PLoS One. 2017 Sep 20;12(9):e0184841. doi: 10.1371/journal.pone.0184841 (PMC5607173; doi:10.1371/journal.pone.0184841)
Supplement: S1 File — Table A in S1 File: Demographics of bladder cancer patients used to assess IL-17 cell infiltration. Table B in S1 File: Function of genes changed in 5637 cells following IL-17 treatment. Table C in S1 File: Detailed histories of BCG-treated CIS patient outcomes and treatment. (DOCX) [file pone.0184841.s003.docx]

Supporting Information

**Table A: Demographics of bladder cancer patients used to assess IL-17 cell infiltration**

| **Sex** | **Male** | **Female** |
| --- | --- | --- |
| Number | 100 ^1^ | 14 |
| **Age (yrs.)^2^** | | |
| Mean (+/- S.D.) | 69.45 (+/- 9.48) | 67.62 (+/-14.44) |
| Minimum | 38.40 | 36.43 |
| Maximum | 89.19 | 86.97 |
| **Stage** | | |
| pTa | 17 | |
| pT1 | 10 | |
| T2+ | 8 | |
| CIS   - *pTis* - *concomitant with pTX* - *concomitant with pT0* - *concomitant with pTa* - *concomitant with pT1* - *concomitant with T2+* | (83)  *3*  *1*  *1*  *18*  *54*  *6* | |
| **Grade** | | |
| 1 | 10 | |
| 2 | 9 | |
| 3 | 95 | |

^1^ The greater number of male patients in the study reflects the higher frequency of bladder cancer in men.

^2^ Age at registration to BCPP, i.e. prior to first treatment/confirmation of diagnosis**.**

**Table B: Function of genes changed in 5637 cells following IL-17 treatment.**

| **Direction of Change** | **Function** | **Number of genes** |
| --- | --- | --- |
|  | Receptor activity | 36 |
|  | Signal transducer activity | 25 |
|  | Receptor activity, Signal transducer activity | 22 |
|  | Receptor activity, G-protein coupled receptor activity, signal transducer activity | 20 |
|  | Response to stimulus | 18 |
|  | Receptor activity, signal transducer activity, response to stimulus | 17 |
|  | Receptor activity, G-protein coupled receptor activity, signal transducer activity, response to stimulus | 16 |
|  | Receptor activity, G-protein coupled receptor activity, signal transducer activity, response to stimulus, olfactory receptor activity | 15 |
|  | Growth factor activity | 10 |
|  | Positive regulation of angiogenesis | 7 |
|  | Cell adhesion | 12 |
|  | Proteolysis | 11 |
|  | Nervous system development | 9 |
|  | Inflammatory response | 8 |
|  | Sequence-specific DNA binding transcription factor activity, positive regulation of transcription, DNA-dependent | 6 |
|  | Sequence-specific DNA binding transcription factor activity, positive regulation of transcription from RNA polymerase II promoter , negative regulation of transcription, DNA-dependent | 4 |
|  | Regulation of transcription, DNA-dependent, sequence-specific DNA binding transcription factor activity, protein binding, positive regulation of transcription, DNA-dependent | 4 |
|  | Ion transmembrane transport, chloride transport | 3 |
|  | Positive regulation of osteoblast differentiation, positive regulation of bone mineralization | 3 |
|  | DNA binding, apoptotic process, sequence-specific DNA binding transcription factor activity, protein binding, positive regulation of transcription from RNA polymerase II promoter , positive regulation of transcription, DNA-dependent | 3 |
|  | Regulation of transcription, DNA-dependent (BP), sequence-specific DNA binding transcription factor activity , protein binding, sequence-specific DNA binding, multicellular organismal development, transcription from RNA polymerase II promoter, positive regulation of transcription, DNA-dependent | 3 |

Genes found to be differentially changed in 5637 cells by IL-17 treatment compared to EJ cells were analysed for co-ordinated functional roles by cluster analysis. Direction of change in 5637 cells, function of these genes and total number of genes changed are shown for each function.

**TableC: Detailed histories of BCG-treated CIS patient outcomes and treatment.**

| **IL-17 count** | **Age** | **Grade** | **Stage** | **Single dose Mytomycin C** | **Induction (doses)^α^** | **Schedule complete^α^** | **Maintenance (doses)^α^** | **Time to Recurrence*** | **Time to Progression*** | **Time to death*** | **COD** | **DLS*** |
| --- | --- | --- | --- | --- | --- | --- | --- | --- | --- | --- | --- | --- |
| 830 | 76.28 | 3 | pTa | Yes | 9 | Yes | 16 |  |  |  |  | 4.26 |
| 813 | 67.57 | 3 | pT1 | Yes | 9 | Yes | Yes |  |  |  |  | 2.84 |
| 775 | 64.33 | 3 | pTa | NK | 9 | Yes | 13 |  |  |  |  | 7.39 |
| 522 | 58.39 | 3 | pT1 | Yes | 9 | Yes | Yes |  |  |  |  | 4.28 |
| 451 | 69.69 | 3 | pTa | Yes | 9 | Yes | 9 |  |  |  |  | 5.85 |
| 414 | 76.33 | 3 | pT1 | Yes | 9 | Yes | Yes |  |  |  |  | 5.35 |
| 374 | 77.17 | 3 | pT1 | NK | 6 | No |  | 0.61 |  |  |  | 0.82^δ^ |
| 352 | 73.00 | 3 | pT1 | Yes | 6 | No | Yes |  |  |  |  | 5.56 |
| 328 | 69.93 | 3 | pT1 | Yes | 9 | Yes | 18 | 0.25 |  |  |  | 4.02 |
| 324 | 77.65 | 3 | pT1 | Yes | NK | - | 16 | 3.56 |  |  |  | 4.13 |
| 317 | 63.78 | 3 | pT1 | Yes | 9 | Yes | 14 |  |  |  |  | 3.76 |
| 288 | 81.78 | 3 | pTis | Yes | 9 | Yes | 0 | 0.59 | 0.91, 1.08 |  |  | 4.09 |
| 286 | 78.89 | 3 | pT1 | NK | 9 | Yes | 18 |  |  |  |  | 7.12 |
| 282 | 69.69 | 3 | pT1 | Yes | 9 | Yes | 17 |  |  |  |  | 3.60 |
| 278 | 59.23 | 3 | pTa | Yes | 5 | No |  | 0.44 |  |  |  | 6.04 |
| 275 | 70.80 | 3 | pT1 | NK | 9 | Yes | 15 | 0.36 |  |  |  | 7.36 |
| 275 | 74.77 | 3 | pT1 | Yes | 9 | Yes | 9 |  |  | 1.83 | Non-BC |  |
| 273 | 65.76 | 3 | pT1 | Yes | 6 | No | Yes | 0.93 |  |  |  | 2.37 |
| 250 | 78.31 | 3 | pTa | NK | 9 | Yes | 9 |  | 0.33, 1.41 | 2.45 | Non-BC |  |
| 226 | 75.31 | 3 | pTa | No | 9 | Yes | Yes |  |  |  |  | 4.08 |
| 223 | 62.13 | 3 | pT1 | Yes | 9 | Yes | 3 | 0.61 | 3.15, 3.63 |  |  | 4.27 |
| 223 | 86.62 | 3 | pT1 | Yes | 6 | No | Yes |  |  | 1.29 | Non-BC |  |
| 222 | 68.27 | 3 | pT1 | Yes | 6 | No | Yes | 0.37 |  | 2.40 | BC |  |
| 209 | 70.34 | 3 | pT1 | Yes | 9 | Yes | 9 |  |  |  |  | 4.36 |
| 189 | 59.08 | 3 | pT1 | NK | 8 | No | 15 |  |  |  |  | 6.41 |
| 188 | 70.90 | 3 | pTa | Yes | 9 | Yes | Yes |  |  |  |  | 4.12 |
| 182 | 81.96 | 3 | pT1 | Yes | 6 | No | No |  |  | 1.56 | BC |  |
| 178 | 77.71 | 3 | pTa | NK | 8 | No | 2 | 1.13, 1.57 |  | 4.56 | Non-BC |  |
| 178 | 77.55 | 3 | pT1 | Yes | 9 | Yes | 5 | 1.02 |  | 4.23 | BC |  |
| 174 | 61.60 | 3 | pTa | NK | 9 | Yes | 17 |  |  |  |  | 8.36 |
| 153 | 75.97 | 3 | pT1 | Yes | 6 | Yes | No |  |  | 3.82 | Non-BC |  |
| 150 | 68.83 | 3 | pT1 | NK | 9 | Yes | 12 |  |  |  |  | 7.74 |
| 142 | 62.83 | 3 | pT1 | Yes | 6 | No | Yes | 1.28 |  |  |  | 1.59^δ^ |
| 141 | 72.62 | 3 | pTa | Yes | 6 | No | No | 0.73 |  |  |  | 3.23^δ^ |
| 136 | 72.20 | 2 | pT1 | NK | 9 | No | 12 |  |  |  |  | 3.83 |
| 132 | 65.17 | 3 | pTa | NK | 9 | Yes | 18 | 0.23, 1.08 |  |  |  | 6.76 |
| 128 | 71.67 | 2 | pT1 | NK | 9 | Yes | 17 |  |  |  |  | 4.75 |
| 120 | 72.50 | 3 | pT1 | Yes | NK |  | NK | 3.28 |  |  |  | 3.30 |
| 117 | 56.05 | 3 | pT1 | Yes | 9 | Yes | Yes |  |  |  |  | 4.08 |
| 110 | 72.00 | 3 | pT1 | Yes | 8 | No | 0 | 3.12 |  |  |  | 6.06 |
| 110 | 80.76 | 2 | pTa | Yes | 9 | Yes | 15 |  |  |  |  | 4.85 |

^α^Course of intravesical Bacillus Calmette Guerin ^δ^Indicates time of cystectomy

*Duration (years) from registration to BCPP, i.e. prior to first treatment/confirmation of diagnosis NK - Unknown
