# Supplementary material for: Interleukin-17-positive mast cells influence outcomes from BCG for patients with CIS: Data from a comprehensive characterisation of the immune microenvironment of urothelial bladder cancer
Source: PLoS One. 2017 Sep 20;12(9):e0184841. doi: 10.1371/journal.pone.0184841 (PMC5607173; doi:10.1371/journal.pone.0184841)
Supplement: S2 File — Table A in S2 File: Details of antibodies used in immunohistochemistry. Table B in S2 File: Details of primers used for quantitative reverse transcriptase PCR. (DOCX) [file pone.0184841.s004.docx]

Supplementary Information - Methods

Patient samples

The present study used samples from the West Midlands Bladder Cancer Prognosis Programme (BCPP), an ongoing multicentre cohort study in the West Midlands, United Kingdom. Details of the study have been published previously [[1](#_ENREF_1)]. Briefly, adult patients (age ≥18 years) presenting with symptoms suspicious of bladder cancer (predominantly haematuria) and referred to one of the participating urology centers within the region were enrolled into the study on the basis of initial findings suggestive of bladder cancer (predominantly abnormal cystoscopy). Those who had a previous diagnosis of cancer of the urethra, bladder, ureter, or renal pelvis within the last decade, HIV infection, or any other condition that might interfere with the ability of the participant to participate fully were excluded. During the enrollment period (19 December 2005-19 January 2011), 2,603 new BC cases were eligible within the catchment areas of whom 1,544 (59%) were recruited. The study protocol was approved by the Nottingham Multicentre Research Ethics Committee (06/MRE04/65), and written informed consent was obtained from all participants. Tumor and tissue samples were snap-frozen in liquid nitrogen in the operating theatre at the time of cystoscopic transurethral resection, and were subsequently stored at -80°C. The diagnostic FFPE tissues were retrieved from local histopathology departments following definitive diagnosis.

IHC Antibodies

FFPE bladder tumor sections were de-waxed in histoclear and rehydrated in increasing concentration of ethanol. Endogenous peroxidise activity was blocked with 0.3% hydrogen peroxide (15min RT) and antigen retrieved by heat induced epitope retrieval (HIER) or protease digestion. Slides were blocked, stained with primary antibody and then secondary antibody using Impress reagents (Vector). Staining was visualised using DAB substrate (Vector) and slides were counterstained with haematoxylin (Sigma) prior to dehydration and mounting. Dual staining was performed with anti-CD3 (DAKO), anti-CD15 (DAKO), anti-CD68 (DAKO) or anti-tryptase (AbD Serotec). Antigen retrieval was performed as described above with the following exceptions; cell linage markers were stained at RT, 1hr, and revealed using Impress anti-Mouse-Ig-HRP (Vector) and DAB substrate. Following first stain, slides were washed, blocked and stained overnight with anti-IL-17 (R&D Systems). IL-17 was detected using the ABC-anti-goat-AP kit (Vector), and Vector Red substrate (Vector). All antibodies were validated and titrated on FFPE human tonsil sections. Additionally to ensure the specificity of the anti-IL-17 antibody binding was blocked using rhIL-17 (peprotech).

**Table B: Details of antibodies used in immunohistochemistry**

| **Antigen** | **Supplier** | **Clone** | **Species** | **Concentration** | **Retrieval** |
| --- | --- | --- | --- | --- | --- |
| IL-17A | R&D systems | AF-317* | Goat | 1/80 | HIER, Citrate pH6 |
| IL-17RA | R&D systems | 133621 | Mouse | 1/100 | HIER, EDTA pH8 |
| CD3 | DAKO | F7.2.38 | Mouse | 1/200 | HIER, EDTA pH8/Citrate pH6 |
| CD15 | DAKO | Carb-3 | Mouse | 1/400 | HIER, EDTA pH8/Citrate pH6 |
| CD68 | DAKO | PG-M1 | Mouse | 1/400 | HIER, EDTA pH8/Citrate pH6 |
| FoxP3 | Abcam | 236A/E7 | Mouse | 1/400 | HIER, Citrate pH6 |
| TCRγ | Santa-Cruz | A-20 | Goat | 1/800 | 0.1% Trypsin 37^o^C |
| ELANE | Santa-Cruz | G-2 | Mouse | 1/200 | HIER, Citrate pH6 |
| Tryptase (MCT) | AbD Serotec | AA1 | Mouse | 1/10000 | HIER, Citrate pH6 |

*polyclonal antibody -catalogue number given

RNA extraction

Cells or tissue were homogenised in Trizol (Invitrogen) using a rotor stator homogenizer. RNA was extracted by addition of 1:5 vol/vol chloroform:Trizol and spun at 13000g, 15min, 4^o^C. Following isolation of RNA in the aqueous phase, an equal volume of EtOH was added and RNA further purified using an RNeasy kit (Qiagen) following the manufacturer’s instructions.

Quantitative RNA analysis

RNA was quantified by q-RTPCR using Taqman probes (Applied Biosystems) listed in Table S1. PCR reactions were prepared in a final volume of 25 µl using Taqman Universal PCR Mastermix (Applied Biosystems). Amplification and detection were performed using an ABI Prism 7500 Sequence Detection System (Applied Biosystems). Thermal cycling conditions comprised an initial incubation (2 min, 50°C) and activation step (10 min, 95°C) followed by 40 rounds of amplification (denaturation (15 s, 95°C), annealing and extension (1 min, 60°C)). All test samples were run in triplicate. Template-negative and control cDNA reactions that lacked reverse transcriptase were included as controls. SDS v1.7 software (Applied Biosystems) was used to determine the Ct value for each sample at which the fluorescence exceeded a threshold value. Ct values are expressed relative to the GAPDH housekeeping gene.

**Table C: Details of primers used for quantitative reverse transcriptase PCR**

| **Gene** | **Assay** | **Label** | **Ref Seq** |
| --- | --- | --- | --- |
| IL-17A | Hs00174383_m1 | FAM | [NM_002190.2](http://www.ncbi.nlm.nih.gov/nuccore/NM_002190.2) |
| IL-17F | Hs00369400_m1 | FAM | [NM_052872.3](http://www.ncbi.nlm.nih.gov/nuccore/NM_052872.3) |
| IL-6 | Hs00985639_m1 | FAM | [NM_000600.3](http://www.ncbi.nlm.nih.gov/nuccore/NM_000600.3) |
| IL-23A p19 | Hs00900828_g1 | FAM | [NM_016584.2](http://www.ncbi.nlm.nih.gov/nuccore/NM_016584.2) |
| GAPDH | 4310884E | VIC/TAMRA |  |

Microarray analysis

EJ or 5637 cells were treated with IL-17 (100ng/ml) as for wounding healing assays;. Extracted RNA integrity (RIN) was assessed on a Agilent 2100 bioanalyzer (Agilent Technologies). A GeneChip Human Genome U133 Plus 2.0 Array was washed and stained on an Affymetrix FS450 fluidics station then scanned using an Affymetrix GeneChip 3000 7G scanner as per Affymetrix procedures. GCOS software (Affymetrix) was used for instrument control and data NM_002046.3acquisition. Changes of 1.5 fold in gene expression were deemed as the cut off point for inclusion. Changes in each cell line were first compared to appropriate mock treated cells. Follow this differential overall change in gene expression between EJ and 5637 cells was also calculated, again a combined change in gene expression of 1.5 fold was used to dictate inclusion. Comparative Analysis and Modular Enrichment Analysis (p values obtained through Hypergeometric analysis corrected by FDR method) were performed using GeneCodis3 [[2](#_ENREF_2)].

1. Zeegers MP, Bryan RT, Langford C, Billingham L, Murray P, et al. (2010) The West Midlands Bladder Cancer Prognosis Programme: rationale and design. BJU International 105: 784-788.

2. Tabas-Madrid D, Nogales-Cadenas R, Pascual-Montano A (2012) GeneCodis3: a non-redundant and modular enrichment analysis tool for functional genomics. Nucleic Acids Research 40: W478-W483.
